# Assessment of Health-Related Quality of Life in Postburn Adult Survivors: A Cross-sectional 4 Years of Experience Study From Cyprus

**DOI:** 10.1093/jbcr/irae213

**Published:** 2025-03-28

**Authors:** Ilias Petrou, Athanasios Papas, Georgina Panopoulou, Andreas Vassiliou

**Affiliations:** School of Medicine, European University Cyprus, Nicosia, Cyprus; School of Medicine, European University Cyprus, Nicosia, Cyprus; Plastic and Reconstructive Surgery Department, Burns Intensive Care Unit, Nicosia General Hospital, Nicosia, Cyprus; Plastic and Reconstructive Surgery Department, Burns Intensive Care Unit, Nicosia General Hospital, Nicosia, Cyprus

**Keywords:** burn injury, quality of life questionnaire, survivors’ perceptions, survivors’ outcomes, self-reported outcomes

## Abstract

Burn injuries have a significant impact on various aspects of survivors’ lives. This study evaluates, assesses, and interprets the health-related quality of life in postburn adult survivors. Of 135 patients admitted from 2019 to 2023 in the burns intensive care unit, 70 responded to a questionnaire. Primary socio-demographic data and details about the cause and extent of burns were collected. A brief EuroQol-5 Dimension-5 Levels (EQ-5D-5L) questionnaire was completed by interviewing burn survivors, and data were then analyzed. The mean age of participants was 52.12 years, and the prevalent gender was males (61.4%). Thermal burns were the most common cause of burn-related injury (91.4%)-most subjects (55.8%) suffered from partial-thickness burns, followed by full-thickness burns affecting 42.8%. The most presented percentage of total body surface area (%TBSA) burned was 1%-10%, accounting for 60% of cases. The most required hospitalization timeframe was 1-5 days. Regarding participants’ quality of life (QoL), most reported no problem in the 5 health dimensions. The health dimensions that were mainly affected were depression/anxiety followed by pain/discomfort. All participants’ mean EQ-visual analogue scale (EQ-VAS) score was 80.98. Our findings disclose a compromised health-related quality of life for postburn individuals, particularly in dimensions of depression/anxiety followed by pain/discomfort. There is a pressing need to establish long-term support for burn survivors by relevant organizations.

## INTRODUCTION

Burn injury is one of the most intense experiences a human being can endure. There is a broad diversity of burn patients regarding age, burn injury mechanisms, site, depth of wound, and comorbidities.^1^ According to the World Health Organization (WHO), 11 million people worldwide suffer from burn injuries and require medical attention. Nearly 180 000 people worldwide have lost their lives to these burns.^2^ The incidence of burn injuries is notably higher in developing countries as opposed to developed ones.^3^ Approximately 90% of burns occur in underdeveloped countries where access to emergency burn care can be limited.^4–6^ Managing severe burns can be challenging, leading to permanent morbidities and an increased mortality rate.^7–10^

The International Society for quality of life research defines the health-related quality of life (HRQoL) as the health aspect of quality of life (QoL) that delves into people’s physical and mental capabilities, their daily functioning, and the extent to which they feel their lives are satisfying and meaningful regarding their health experiences.^11^ HRQoL is a commonly studied outcome after burn injuries, both in the short and long term. Survivors of major burn injuries often report restrictions in many aspects of their daily lives, such as physical, psychological, emotional, social, and environmental dimensions. Consequences can be varied, including but not limited to restricted physical activity, posttraumatic stress disorder (PTSD), and depression/anxiety.^1,12–21^

Different tools have been developed for the assessment of HRQoL in postburn survivors, with the most used being the Burn Specific Health Scale Brief (BSHS), the Medical Outcomes Survey Short Form-36 (SF-36), and the EuroQol-5 Dimension (EQ-5D).^22^ This study employed the EQ-5D, version EuroQol-5 Dimension-5 Levels (EQ-5D-5L). The EQ-5D-5L questionnaire is a relatively brief and straightforward tool. It makes it easy for participants to understand and complete as it comes with only 5 dimensions and a simple, intuitive 5-level Likert scale to rate each dimension. Furthermore, this study primarily aims to evaluate the HRQoL in burn survivors. The EQ-5D-5L questionnaire has been designed to assess HRQoL across the 5 dimensions, all of which are relevant to the experiences of burn survivors. Finally, it includes a combination of a descriptive system and a visual analog scale, offering a comprehensive way to measure and interpret patients’ self-reported health status.

The 5 levels of EQ-5D were introduced by the EuroQol Foundation to enhance the instrument’s sensitivity and to reduce ceiling effects compared to the EuroQol-5 Dimension-3 Levels (EQ-5D-3L). The EQ-5D-5L comprises the EQ-5D-5L descriptive system and the EQ-Visual Analogue Scale (EQ-VAS).^23^ It covers 5 health dimensions (mobility, self-care, usual activities, pain/discomfort, and anxiety/depression) with 5 levels of severity (no problems, slight problems, moderate problems, severe problems, and unable to/extreme issues) in each of the existing 5 dimensions.^23^ Furthermore, the EQ-VAS is designed to record participant’s overall current health on a vertical visual analogue scale, with the endpoints labelled as “The best health you can imagine” (indicated with number 100) and “The worst health you can imagine” (indicated with number 0). The EQ-VAS dispenses a quantitative measure of the participant’s perception of their overall health.^23^

Gautam et al.^18^ stated in their cross-sectional study on HRQoL in postburn survivors that the psychological and physical domains were the most affected. Meanwhile, the environmental and social domains were the least affected. As reported by Tibebu et al.^19^ in their study, 57.5% of participants (total number 405) had a poor postburn QoL, especially those with third-degree burns, exposed burn body parts, total body surface area (%TBSA) burned ≥ 20%, amputation and comorbidities. Two studies have also found a diminished postburn QoL.^20,21^ Specifically, the authors concluded that postburn hospital discharge had caused significant physical and psychological impairment over time, along with emotional and social consequences.^20^

Egyhazi et al.^24^ stated in their study on chronic pain following physical and emotional trauma in burn survivors that 27% of participants reported pain at least 28 months after the incident. Several factors related to pain were evaluated in the univariate analysis; however, only age, graft, and back depression inventory score remained significantly associated with pain in the multiple regression model. According to a systematic review and meta-analysis on prevalence and predictors of posttraumatic stress symptomatology among burn survivors, the baseline prevalence of acute stress disorder ranged from 2% to 30%. In comparison, PTSD prevalence varied from 3% at 1 month to 40% by 6 months, peaking at 45% within a year postinjury and settling between 7% and 25% after 2 years. Life threat perception emerged as the strongest predictor of PTSD, followed by acute intrusive symptoms and pain related to burn injuries.^25^

Additionally, 2 studies conducted in the United States analyzed postburn itchiness status, medically termed pruritus, in both pediatric and adult populations, respectively.^26,27^ The Itch Man Scale demonstrated strong reliability in the pediatric population, with independent rater scores showing a high correlation. It also showed a significant correlation with the visual analog scale, a standard pruritus measure. Additionally, the Itch Man Scale correlated with the total score of the 5-D Itch scale, though only the degree domain individually reached a significant correlation.^26^ An additional study by Carrougher et al.^27^ examines the postburn prevalence and risk factors associated with increased pruritus intensity in adults. They found a pruritus prevalence at discharge, 6, 12, and 24 months following injury at 93%, 86%, 83%, and 73%, respectively. Risk factors associated with increased pruritus intensity include younger age, higher %TBSA-burn, higher %TBSA-grafted, and female gender.

Some crucial predictors of morbidity and mortality for burn patients include gender, %TBSA, length of hospital stay (LOS), the necessity for surgical treatment, pain, and the psychological impact of burns.^21,28–30^ According to Spronk et al.^30^ systematic review of HRQoL after burns, male burn patients had better HRQoL than females. Five studies found lower HRQoL in more severely burned patients, while longer LOS was associated with poorer HRQoL. Burns leading to surgery and pain were also reported as predictors for diminished HRQoL. Lastly, research on postburn depression was positively associated with impaired HRQoL.^30^

The study hypothesises that our country’s burn survivors exhibit district patterns in HRQoL. The study population may experience more significant challenges in specific areas due to the socioeconomic and healthcare access factors that impact the postinjury recovery experience. Specifically, socioeconomic factors such as limited access to comprehensive rehabilitation, including pain management, physiotherapy, psychological support, or social reintegration programs, may lead to higher levels of physical discomfort/pain, limited mobility and diminished emotional well-being. We anticipate that these barriers will be reflected in worse functioning scores, as survivors may not receive the ongoing care needed to optimise recovery.

This study aimed to evaluate, assess, and interpret the HRQoL of our country’s postburn adult survivors. It also aimed to establish insights into their general health-related condition, enabling appropriate organizations to provide personalised ongoing health care.

## MATERIALS AND METHODS

### Materials

#### Study design, setting, and participants

This cross-sectional study was conducted in our hospital’s burns outpatient clinic; of the 135 adult patients previously treated in the hospital’s burns intensive care unit, the country’s single burn care centre, 70 (52%) responded to the questionnaire.

#### Inclusion and exclusion criteria

This study included burn survivors aged over 18 years, with injuries ranging from minor (<10% of TBSA with predominantly superficial burns) to severe (>10% TBSA in elderly patients and more than 20% in adult patients). Critical issues such as inhalation injury, comorbidities/complications, young/elderly patients, complex wounds require care, surgical intervention, and pain management to warrant intensive care unit admission. Participants sustained burns of any kind, including thermal, chemical, electrical, and friction burns, and the time passed from the day of admission to the interview was mainly more than 1 year to up to 4 years postburn. The study excluded individuals with severe disabilities, as well as those with communication, vision, and hearing disorders, along with cases involving homicidal or suicidal burns.

#### Primary and secondary outcomes

The study’s primary outcomes are the assessment of HRQoL in postburn adult survivors and the influence of burn injury characteristics (extent, depth, and type) on the quality of life of burn survivors. The study’s secondary outcomes include identifying the essential socio-demographic characteristics and burn-related clinical parameters within the study population. Additionally, it will be examined how age and gender affect the quality of life of burn survivors. Moreover, it will explore how burn injuries affect various aspects of survivors’ daily lives, including mobility, self-care, usual activities, pain/discomfort, and anxiety/depression. Lastly, it will be investigated whether the effects of burn injuries on QoL are evenly distributed among survivors.

#### Study tool

The total number of participants, mean age at the time of injury, gender, type of burn injury, extent, depth, and hospital LOS were recorded. The HRQoL assessment was conducted through a structured interview using the EQ-5D, version 5Q-5D-5L (Appendix 1).

### Methods

Data from participants’ medical records at our hospital’s Plastic and Reconstructive Surgery Department were collected in September 2023 using Microsoft Excel.^31^ All participants met the study’s inclusion criteria, and follow-up interviews were conducted weekly from October to December 2023, each lasting approximately 25 min. The interviewer managed participant appointments, explained the study and questionnaire structure, collected consent forms, and answered any participant questions. The EQ-5D-5L scores ranged from 1 (no problems) to 5 (severe impairment) across health domains, and participants’ self-rated health was recorded using the EQ-VAS.

#### Patient’s consent

After the authors explained the study’s purpose and nature, the subjects who agreed to participate provided written informed consent. The authors ensured the participants’ anonymity and confidentiality.

#### Ethical considerations

In July 2023, this study was submitted for approval to the National Bioethics Committee and the Research and Innovation office of the state Health Services organization. Official approval was granted in September 2023.

#### Statistical analysis

Statistical analysis was performed using the Statistical Package for Social Sciences. Descriptive statistics (mean ± SD) were employed to express continuous variables. In contrast, frequencies and percentages were used for categorical variables. Using cross-tabulations, the association between 2 or more variables was evaluated. Chi-square statistics were then employed to examine univariate associations between these variables. A *P*-value < .05 was considered statistically significant.

## Results

Out of 135 patients who were admitted, 70 responded to a questionnaire and met the inclusion criteria. The current study reported the following parameters.

### Sample characteristics

**Figure 1.**
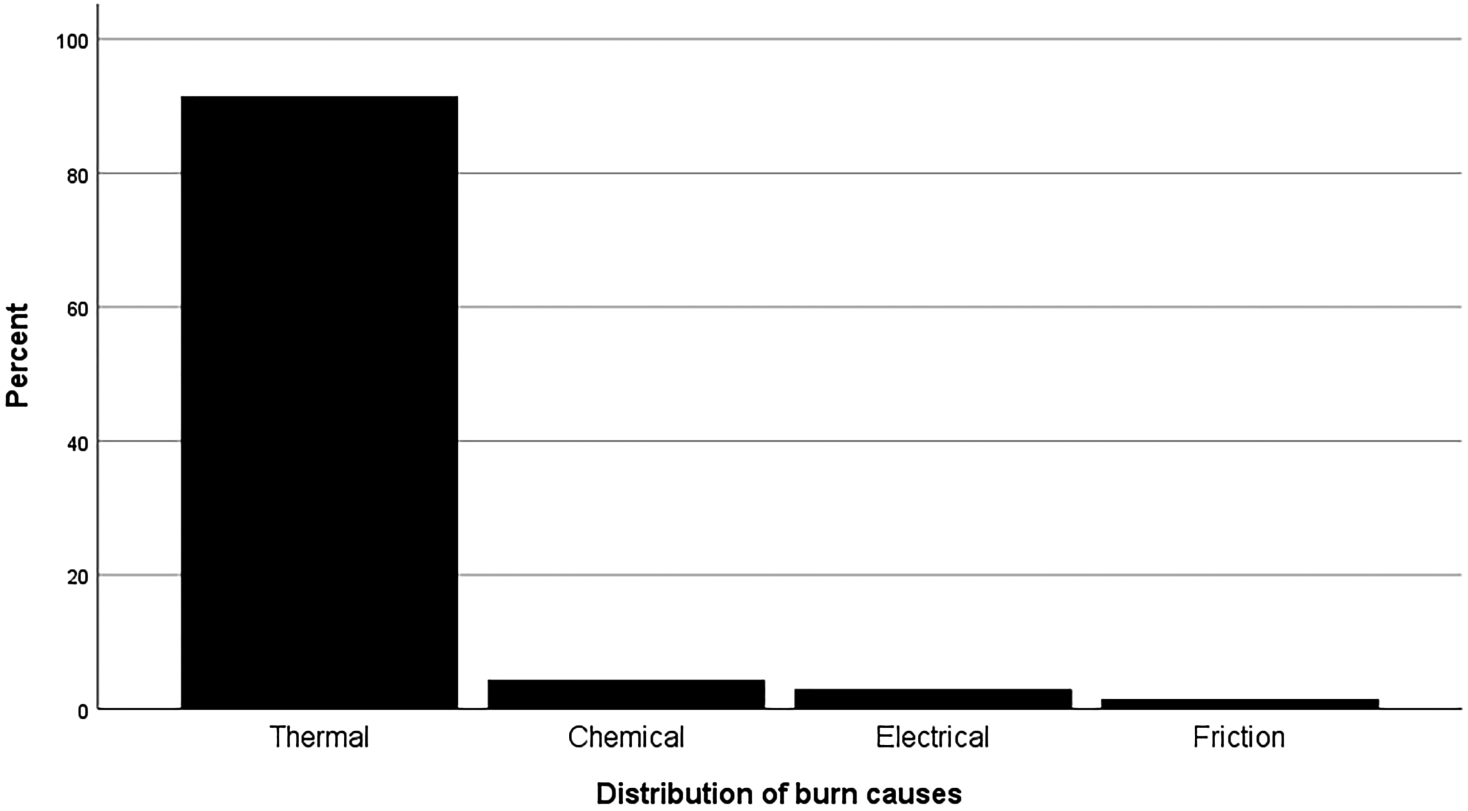
Percent Distribution of Burn Causes in Adult Survivors

Table 1 provides the essential socio-demographic characteristics of the study population. The mean age of participants was 52.12 years, with the most frequent age group being 56-65. The majority were male (61.4%). Thermal burns were the most common cause of burn injury (91.4%), while friction burns were the least frequent (1.4%) (Figure 1). Most participants had partial-thickness burns (55.8%) and burns covering 1%-10% TBSA (60%). For most (80%), over a year had passed since the injury, and the most common hospitalization duration was 1-5 days (30%). In Supplementary Table 1, cross-tabulations illustrate the association between burn etiology and gender/age. They revealed no statistically significant association between burn etiology and gender (*P*-value = .409) or age (*P*-value = .341).

**Table 1.** Participants’ Essential Socio-demographic Characteristics and Characteristics According to Burn’s Parameters

Variable	Frequency (*N*)	Percent (%)
**Age in categories (years)**		
18-25	5	7.1
26-35	13	18.5
36-45	9	12.8
46-55	10	14.2
56-65	16	22.8
66-75	8	11.3
76-85	9	12.7
**Gender**		
Male	43	61.4
Female	27	38.6
Total	70	100.0
**Parameter**	**Frequency (*N*)**	**Percent (%)**
**Degree of burn**		
Superficial—thickness (first degree)	1	1.4
Partial—thickness (second degree)	39	55.8
Full—thickness (third degree)	30	42.8
%TBSA burned*		
1-10	42	60.0
11-20	16	22.8
21-30	7	10.0
31-40	2	2.9
41-50	2	2.9
51-60	1	1.4
**Time since burn**		
<1 year	14	20.0
>1 year	56	80.0
**Hospitalization time (days)**		
1-5	21	30.0
6-10	17	24.3
11-15	13	18.6
16-20	7	10.0
21-25	2	2.9
26-30	1	1.4
31-35	4	5.7
36-40	1	1.4
46-50	2	2.9
51-55	1	1.4
56-60	1	1.4
**Total**	70	100.0

### Descriptive statistics of health-related quality of life in postburn adult survivors

Using the EQ-5D-5L questionnaire, most participants reported no problems in the 5 health dimensions: depression/anxiety (36/70) and pain/discomfort (32/70) were the most affected areas, while mobility and self-care were the least affected, with 63 and 57 participants reporting no issues, respectively (Table 2).

**Table 2.** EQ-5D-5L Frequencies and Proportions are Reported by Dimension and Level

EQ-5D	Responses (*N*/%)				
Dimensions	No problems	Slight problems	Moderate problems	Severe problems	Unable to/extremely pain/extremely anxious
Mobility	63 (90.0)	4 (5.7)	2 (2.9)	1 (1.4)	0
Self-care	57 (81.4)	7 (10.0)	4 (5.7)	2 (2.9)	0
Usual activities	52 (74.3)	8 (11.4)	7 (10.0)	3 (4.3)	0
Pain/discomfort	38 (54.3)	20 (28.6)	9 (12.9)	2 (2.9)	1 (1.4)
Depression/anxiety	34 (48.6)	16 (22.4)	15 (21.4)	4 (5.7)	1 (1.4)

### Cross-tabulation analysis of ED-5D-5L dimensions according to participants’ age and gender, as well as extent of %TBSA and burn depth

Supplementary Tables 2 and 3 illustrate no significant association between ED-5D-5L dimensions and participants’ age and gender. However, older participants reported more problems across all dimensions (Supplementary Table 2). Supplementary Tables 4 and 5 indicate no significant associations between ED-5D-5L dimensions and %TBSA and burn depth, except for mobility (*P*-value = .046) and usual activities (*P*-value = .037), which were significantly associated with burn depth.

### Total EQ-5D-5L and EQ-VAS scores of all participants after treatment

**Figure 2.**
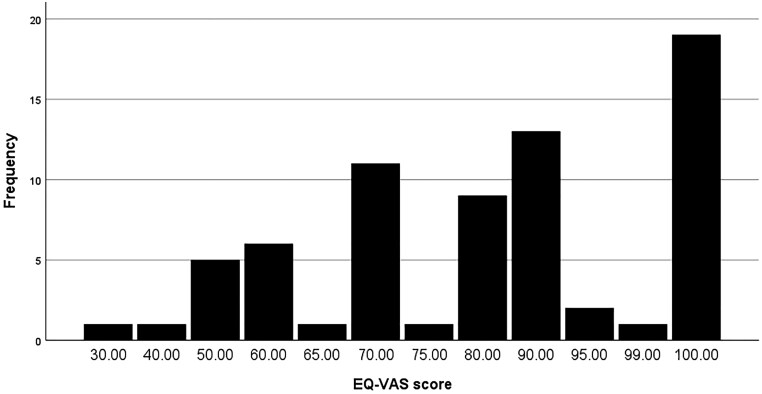
Frequency Distribution of EQV-VAS Score

Table 3 shows that the median EQ-5D-5L scores across all 5 health domains were one, indicating an uncompromised health-related condition. The most severe score of 5, indicating extreme problems, was only reported in anxiety/depression and pain/discomfort. The mean EQ-VAS scores were 80.98, with a median of 85 (Table 3). Scores ranged from 30 to 100 (Figure 2). Notably, 19 participants scored 100%, and 13 scored 90%. The 76-85-year-old age group reported the lowest scores, while the highest scores were reported by those aged 26-35.

**Table 3.** EQ-5D -5L Scores and EQ-VAS Values by Age—Mean + SD and Median After Treatment

EQ-5D-5L	Mobility	Self-care	Activity	Pain/discomfort	Anxiety/depression			
**Mean**	1.15	1.30	1.44	1.68	1.88			
**Std error**	0.063	0.084	0.101	0.108	0.123			
**Median**	1.00	1.00	1.00	1.00	1.00			
**Minimum**	1.00	1.00	1.00	1.00	1.00			
**Maximum**	4.00	4.00	4.00	5.00	5.00			
***N***	70	70	70	70	70			
**EQ VAS**								
**Age groups**	**18-25**	**26-35**	**36-45**	**46-55**	**56-65**	**66-75**	**76-85**	**Total**
**Mean**	78.00	91.07	85.55	76.50	79.00	83.33	69.44	80.98
**Std Dev**	21.67	14.97	11.30	21.60	17.13	19.36	15.89	17.85
**Median**	70.0	100.00	90.00	75.00	90.00	90.00	70.00	85.00

## Discussion

Burn injury is a significant global health concern, leading to increased morbidity, mortality, and a decrease in QoL among survivors.^8–10^ This study aims to evaluate, assess, and interpret the HRQoL of postburn adult survivors in our country, utilizing the standardized EQ-5D-5L questionnaire. By understanding the overall health-related conditions of burn survivors, relevant organizations could provide personalized healthcare that addresses physical and mental needs.

In this study, the calculated mean age of participants was 52.12 years, comparable to findings from other studies,^32,33^ suggesting a similar demographic trend in burn injury populations. Understanding the average age of burn survivors is crucial for designing age-appropriate prevention and intervention strategies, as different age groups may have varying risks and recovery challenges. Furthermore, the study found that males were the most commonly affected gender, which aligns with the general burn epidemiology. This trend is also observed in the findings of Ali et al.^34^ who reported that 56.6% of burn survivors were males. This gender disparity in burn incidence could be attributed to occupational hazards and greater exposure to high-risk environments, highlighting the need for targeted safety measures and awareness campaigns directed explicitly at men.

Burn injuries were classified into thermal, chemical, electrical and friction burns, with thermal burns being the most prevalent type among participants. This predominance of thermal burns is significant because it highlights the need for targeted prevention strategies, such as fire safety education and improved safety standards in environments prone to heat exposure. This finding aligns with the retrospective study by Toolaroud et al.^35^ where fire and flame injuries were identified as the most potent, suggesting a consistent pattern across different populations.

Moreover, the cross-tabulation analysis revealed an insignificant association between burn etiology and the gender/age of participants (Supplementary Table 1), indicating that the cause of burns affects individuals across all demographic groups equally. This observation supports the research conducted by Shahid et al.^36^ which similarly found a statistically nonsignificant correlation between burn etiology and demographic factors. This study emphasizes the universal nature of burn risks and the need for comprehensive prevention and education efforts across all age groups and genders.

Burn types and sizes are crucial factors influencing patient recovery and healthcare needs. In our study, most participants suffered from partial-thickness burns, which typically require extensive wound management and can lead to complications such as scarring and contractures if not adequately treated. Full-thickness burns, while less common, present an even more significant challenge, often necessitating surgical intervention, including skin grafts. These findings are consistent with the results of Alajmi et al.^37^ reporting second-degree burns as the most frequent (71.1%) followed by third-degree burns (16.1%). This similarity underscores the importance of developing protocols focused on managing partial-thickness injuries, as they represent most cases.

Additionally, in the present study, the majority of patients presented with burns ranging from 1% to 10% of their TBSA. This pattern mirrors findings from Abarca et al.^38^ who observed a mean TBSA area of 8.3%. For instance, more extensive TBSA burns typically result in prolonged hospital stays and increased risk of complications. The relatively small uncomplicated burn sizes are significant because they suggest that many patients may benefit from outpatient treatment, although even minor burns can have substantial physical and psychological impacts. Understanding the distribution of burn sizes can help allocate healthcare resources effectively, emphasizing early intervention, comprehensive burn care, and follow-up services to improve patient outcomes.

Most participants reported no problems with their quality of life in the 5 health dimensions. The health dimensions most affected were depression/anxiety and pain/discomfort. These findings highlight the significant long-term psychological and physical impact that burn injuries can have, even when physical wounds have healed. Spronk et al.^30^ systematic review supports this observation, demonstrating that recovery in the physical (pain) and emotional domains (depression and anxiety) is often prolonged and incomplete compared to other areas of health. This emphasizes the need for comprehensive rehabilitation that addresses both mental and physical health challenges postinjury.

Conversely, mobility and self-care were the least affected dimensions in this study, suggesting that many survivors could maintain or regain functional independence. This underscores the importance of prioritizing pain management and psychological support in burn aftercare, as physical functionality may recover more quickly than emotional well-being and chronic pain issues. Addressing these aspects holistically could significantly enhance burn survivors’ overall quality of life, facilitating a more complete, and balanced recovery.

Cross-tabulation tables (Supplementary Tables 2 and 3) showed a statistically insignificant association between the ED-5D-5L dimensions and participants’ age and gender. Generally, as participants’ ages advanced, more problems were reported in each of the 5 dimensions. There are a variety of potential reasons to explain this finding. Firstly, even though patients with severe disabilities or communication issues were excluded to help focus on a specific patient group, they do not eliminate the complexities associated with ageing. Older patients might still experience higher levels of frailty, which could lead to a greater likelihood of reporting problems. Furthermore, even in the absence of severe disabilities, older adults are more likely to have chronic health conditions that could exacerbate their experience of health problems. Lastly, psychological or social factors such as loneliness or fear, which might be more prevalent in older populations, could influence their perception and reporting of problems.

Nonsignificant associations between ED-5D-5L dimensions and participants’ extent of %TBSA are illustrated in Supplementary Table 4. These findings are incompatible with the results of the study conducted by Schahid et al.^36^ who demonstrated a significant association between all the ED-5D-5L dimensions and participants’ extent of %TBSA. The differences between our findings and those of Schahid et al. may be attributed to several important distinctions in the study populations and burn characteristics. Our cohort consisted primarily of patients with relatively small burns, with most cases being less than 10% TBSA. On the other hand, Schahid et al.’s study included a higher proportion of patients with larger burns, 11%-20% TBSA, which could explain the stronger association they found between the EQ-5D-5L dimensions and the extent of %TBSA.

Mobility and usual activities were the only domains that showed a statistically significant association between EQ-5D-5L dimensions and subjects’ degree of burn (Supplementary Table 5). This finding underscores the profound impact that the depth of burn injuries can have on a person’s physical functioning and daily life. Participants with full-thickness burns, for instance, reported more severe mobility issues, ranging from moderate to severe difficulties, compared to those with superficial or partial-thickness burns, who generally reported no problems in these domains. This finding may reflect the physical limitations or complications that tend to increase as the depth and severity of burns increase.

Additionally, the ability to perform daily activities varies significantly with the degree of burn severity. Those with full-thickness or more severe burns were more likely to report moderate to severe problems, while those with superficial burns reported fewer issues. This could be associated with the increased rehabilitation demands, pain, or physical impairments that prevent survivors with severe burns from resuming their everyday activities. However, in the study by Schahid et al.^36^ mobility had an insignificant association with the degree of burn. At the same time, usual activities were positively associated with the burn degree, as observed in our study.^36^

The association between deeper burns and greater mobility impairment highlights the need for tailored rehabilitation programs that address the long-term physical challenges faced by these patients. Early physical therapy and interventions to prevent contractures and promote mobility could be critical in improving outcomes. Furthermore, the impact on usual activities underscores how significant burn injuries can disrupt daily living and occupational functioning, emphasizing the importance of comprehensive care plans that support patients’ return to normal life activities. Healthcare providers can better assist patients in achieving optimal recovery and functional independence by focusing resources on those with deeper burns.

Median EQ-5D-5L scores for all 5 domains after treatment were calculated at one, reflecting the best possible health state. This suggests that, overall, participants perceived their quality of life as fully restored in terms of mobility, self-care, usual activities, pain/discomfort, and anxiety/depression following treatment. However, when evaluating the mean EQ-VAS score, participants reported an average of 80.98, which, while high, indicates some residual health concerns. This finding aligns closely with the additional study by Spronk et al.^39^ which reported a similar mean EQ-VAS score of 81.60, emphasizing a generally positive but not perfect perception of health after recovery from burn injuries.

Interestingly, age played a significant role in reported health outcomes. The age group of 76-85 years reported the lowest EQ-VAS scores, highlighting that older patients may face greater challenges in fully recovering or perceiving their health as optimal after burn treatment. This is consistent with findings from Spronk et al.^40^ who identified older age at the time of injury as a predictor of lower EQ-VAS scores, likely due to factors such as slower healing, preexisting health conditions, and greater physical frailty. In contrast, the 26-35 age group reported the highest EQ-VAS scores, suggesting that younger adults experience better recovery outcomes and a higher perceived quality of life after treatment. This age-related disparity emphasizes the importance of age-specific interventions and support strategies to optimize recovery and improve health perceptions among older burn survivors.

Summarily, this cross-sectional study reveals that postburn individuals experience compromised health-related quality of life, especially in the areas of depression/anxiety and pain/discomfort. By understanding which HRQoL aspects are most affected, we can adjust care protocols to prioritize the psychological and physical needs that are most relevant to our population. This can foster more efficient resource allocation, inform policymakers, and lead to the development of specialized rehabilitation programs that directly address the challenges faced by burn survivors.

### Limitations

While our study provides valuable insights into the postburn health-related condition of survivors, it is essential to acknowledge some limitations. First, the study relied on patients’ self-reported outcomes, which may be subject to reporting bias. Participants may have overestimated or underestimated their symptoms due to social desirability bias or response bias. To address this concern, we have taken several steps to enhance the rigour and reliability of our analysis. Strategies to address reporting bias included: (a) utilizing a validated measurement tool, (b) ensuring confidentiality and anonymity, (c) minimizing leading questions, and lastly, (d) building rapport and trust with participants. Rapport and trust were achieved by creating a comfortable interview setting, providing a clear explanation of the purpose of the study, practising active listening, avoiding interruptions, using clear and straightforward language.

Additionally, it is fundamental to acknowledge the fact that most of our study’s participants had suffered from thermal partial and full-thickness burns and burns ranging from 1% to 10% TBSA. This distribution encompasses a range of burn severities, albeit it might not include extreme cases. Therefore, while our findings offer insights into the experiences and health outcomes of patients with mainly partial and full-thickness burns, the applicability to those with more severe or complex burn injuries may be limited. To address this limitation and enhance the generalizability of future studies, we recommend stratifying participants based on burn mechanism and severity.

Furthermore, we recognize the absence of control groups as a potential limitation of our study, which could lead to confounding bias. While control groups are commonly used in experimental research to establish causal relationships, our research employed an observational study, which limits our ability to control for confounding variables. Lastly, it is essential to acknowledge that almost half of the patients were excluded from the study, some due to unresponsiveness and others because they needed to fulfil the eligibility criteria.

## Conclusion

Understanding postburn survivors’ health-related quality of life is fundamental for providing comprehensive care and support. This cross-sectional study reveals a compromised health-related quality of life for postburn individuals, particularly in dimensions of depression/anxiety followed by pain/discomfort. There is a pressing need for the establishment of long-term physical and psychosocial support for burn survivors by relevant organizations.

## Supplementary Material

irae213_suppl_Supplementary_Appendix

irae213_suppl_Supplementary_Tables
