# Supplementary material for: Assessment of Health-Related Quality of Life in Postburn Adult Survivors: A Cross-sectional 4 Years of Experience Study From Cyprus
Source: J Burn Care Res. 2025 Mar 28;46(4):768–75. doi: 10.1093/jbcr/irae213 (PMC12397683; doi:10.1093/jbcr/irae213)
Supplement: irae213_suppl_Supplementary_Tables [file irae213_suppl_supplementary_tables.zip › Supplementary Tables/Supplementary Tables.pdf]

Supplementary Table 1. *The cross-tabulation table presents the burn aetiology distribution (count and %) according to the participant's gender and age.*

			Age (years)									Gender			
			**18-25**	**26-35**	**36-45**	**46-55**	**56-65**	**66-75**	**76-85**	**Total**	**p-value**	**Male**	**Female**	**Total**	**p-value**
**Burn etiology**	Thermal	Count	5	12	7	9	15	7	9	64	.341	39	25	64	.409
		% within Age	100.0	92.3	77.8	90.0	100.0	77.8	100.0	91.4		90.7	92.6	91.4	
	Chemical	Count	0	0	1	0	0	2	0	3		2	1	3	
		% within Age	0.0	0.0	11.1	0.0	0.0	22.2	0.0	4.3		4.7	3.7	4.3	
	Electrical	Count	0	0	1	1	0	0	0	2		2	0	2	
		% within Age	0.0	0.0	11.1	10	0.0	0.0	0.0	2.9		4.7	0.0	2.9	
	Friction	Count	0	1	0	0	0	0	0	1		0	1	1	
		% within Age	0.0	7.7	0.0	0.0	0.0	0.0	0.0	1.4		0.0	3.7	1.4	
**Total**		Count	5	13	9	10	15	9	9	70		47	27	70	
		% within Age	100.0	100.0	100.0	100.0	100.0	100.0	100.0	100.0		100.0	100.0	100.0	

Supplementary Table 2. *Cross-tabulation of EQ-5D dimensions (Mobility, Self-care, Usual Activities, Pain/Discomfort, Anxiety/Depression) according to age group (Years). The table presents participants' distribution (count and percentage) reporting levels 1 to 5 for each dimension across different age categories. Percentages are calculated within each age group, and the total number of participants is 70.*

**EQ-5D**	**Age categories (years)**								
**Dimension**	**18-25**	**26-35**	**36-45**	**46-55**	**56-65**	**66-75**	**76-85**	**Total**	**p-value**
**Mobility N/(%)**									
No problems	5 (100.0)	13 (100.0)	9 (100.0)	9 (90.0)	13 (86.7)	7 (77.8)	7 (77.8)	63 (90.0)	0.448
Slight problems	0 (0.0)	0 (0.0)	0 (0.0)	0 (0.0)	1 (6.7)	2 (22.2)	1 (11.1)	4 (5.7)	
Moderate problems	0 (0.0)	0 (0.0)	0 (0.0)	1 (10.0)	1 (6.7)	0 (0.0)	0 (0.0)	2 (2.9)	
Severe problems	0 (0.0)	0 (0.0)	0 (0.0)	0 (0.0)	0 (0.0)	0 (0.0)	1 (11.1)	1 (1.4)	
Unable to	0 (0.0)	0 (0.0)	0 (0.0)	0 (0.0)	0 (0.0)	0 (0.0)	0 (0.0)	0 (0.0)	
**Self-care N/(%)**									
No problems	4 (80.0)	13 (100.0)	8 (88.9)	9 (90.0)	12 (80.0)	6 (66.7)	5 (55.6)	57 (81.4)	0.440
Slight problems	0 (0.0)	0 (0.0)	1 (11.1)	1 (10.0)	1 (6.7)	2 (22.2)	2 (22.2)	7 (10.0)	
Moderate problems	1 (20.0)	0 (0.0)	0 (0.0)	0 (0.0)	2 (13.3)	0 (0.0)	1 (11.1)	4 (5.7)	
Severe problems	0 (0.0)	0 (0.0)	0 (0.0)	0 (0.0)	0 (0.0)	1 (11.1)	1 (11.1)	2 (2.9)	
Unable to	0 (0.0)	0 (0.0)	0 (0.0)	0 (0.0)	0 (0.0)	0 (0.0)	0 (0.0)	0 (0.0)	
**Usual activities N/(%)**									
No problems	4 (80.0)	12 (92.3)	8 (88.9)	7 (70.0)	12 (80.0)	5 (55.6)	4 (44.4)	52 (74.3)	0.080
Slight problems	0 (0.0)	1 (7.7)	0 (0.0)	1 (10.0)	1 (6.7)	1 (11.1)	4 (44.4)	8 (11.4)	
Moderate problems	1 (20.0)	0 (0.0)	1 (11.1)	2 (20.0)	2 (13.3)	1 (11.1)	0 (0.0)	7 (10.0)	
Severe problems	0 (0.0)	0 (0.0)	0 (0.0)	0 (0.0)	0 (0.0)	2 (22.2)	1 (11.1)	3 (4.3)	
Unable to	0 (0.0)	0 (0.0)	0 (0.0)	0 (0.0)	0 (0.0)	0 (0.0)	0 (0.0)	0 (0.0)	
**Pain/Discomfort N/(%)**									
No pain	3 (60)	9 (69.2)	5 (55.6)	3 (30.0)	10 (66.7)	2 (22.2)	6 (66.7)	38 (54.3)	0.295
Slight pain	0 (0.0)	3 (23.1)	2 (22.2)	5 (50.0)	4 (26.7)	5 (55.6)	1 (11.1)	20 (28.6)	
Moderate pain	2 (40.0)	1 (7.7)	1 (11.1)	2 (20.0)	1 (6.7)	1 (11.1)	1 (11.1)	9 (12.9)	
Severe pain	0 (0.0)	0 (0.0)	1 (11.1)	0 (0.0)	0 (0.0)	1 (11.1)	0 (0.0)	2 (2.9)	
Extremely pain	0 (0.0)	0 (0.0)	0 (0.0)	0 (0.0)	0 (0.0)	0 (0.0)	1 (11.1)	1 (1.4)	
**Anxiety/Depression N/(%)**									
Non anxious/depressed	2 (40.0)	10 (76.9)	6 (66.7)	4 (40.0)	7 (46.7)	2 (22.2)	3 (33.3)	34 (48.6)	0.354
Slight anxious/depressed	1 (20.0)	2 (15.4)	2 (22.2)	2 (20.0)	3 (20.0)	2 (22.2)	4 (44.4)	16 (22.9)	
Moderately anxious/depressed	1 (20.0)	1 (7.7)	1 (11.1)	3 (30.0)	5 (33.3)	4 (4.44)	0 (0.0)	15 (21.4)	
Severely anxious/depressed	1 (20.0)	0 (0.0)	0 (0.0)	1 (10.0)	0 (0.0)	1 (11.1)	1 (11.1)	4 (5.7)	
Extremely anxious/depressed	0 (0.0)	0 (0.0)	0 (0.0)	0 (0.0)	0 (0.0)	0 (0.0)	1 (11.1)	1 (1.4)	
**Total**									
**Count**	5	13	9	10	15	9	9	70	
**% within Age**	100.0	100.0	100.0	100.0	100.0	100.0	100.0	100.0	

Supplementary Table 3. *Cross-tabulation of EQ-5D dimensions (Mobility, Self-care, Usual Activities, Pain/Discomfort, Anxiety/Depression) according to gender. The table presents participants' distribution (count and percentage) of male and female responders reporting levels 1 to 5 for each dimension. Percentages are calculated within each gender group, with a total sample size of 70 participants (43 males, 27 females).*

**EQ-5D**	**Gender**			
**Dimension**	**Male**	**Female**	**Total**	**p-value**
**Mobility N/(%)**				
No problems	40 (63.5)	23 (36.5)	63 (100.0)	0.158
Slight problems	3 (75.0)	1 (25.0)	4 (100.0)	
Moderate problems	0 (0.0)	2 (100.0)	2 (100.0)	
Severe problems	0 (0.0)	1 (100.0)	1 (100.0)	
Unable to	0 (0.0)	0 (0.0)	0 (0.0)	
**Self-care N/(%)**				
No problems	36 (63.2)	21 (36.8)	57 (100.0)	0.440
Slight problems	5 (71.4)	2 (28.6)	7 (100.0)	
Moderate problems	1 (25.0)	3 (75.0)	4 (100.0)	
Severe problems	1 (50.0)	1 (50.0)	2 (100.0)	
Unable to	0 (0.0)	0 (0.0)	0 (0.0)	
**Usual activities N/(%)**				
No problems	33 (63.5)	19 (36.5)	52 (100.0)	0.609
Slight problems	4 (50.0)	4 (50.0)	8 (100.0)	
Moderate problems	5 (71.4)	2 (28.6)	7 (100.0)	
Severe problems	1 (33.3)	2 (66.7)	3 (100.0)	
Unable to	0 (0.0)	0 (0.0)	0 (0.0)	
**Pain/Discomfort N/(%)**				
No pain	24 (63.2)	14 (36.8)	38 (100.0)	0.233
Slight pain	14 (70.0)	6 (30.0)	20 (100.0)	
Moderate pain	5 (55.6)	4 (44.4)	9 (100.0)	
Severe pain	0 (0.0)	2 (100.0)	2 (100.0)	
Extremely pain	0 (0.0)	1 (100.0)	1 (100.0)	
**Depression/Anxiety N/(%)**				
Non anxious/depressed	24 (70.6)	10 (29.4)	34 (100.0)	0.418
Slight anxious/depressed	8 (50.0)	8 (50.0)	16 (100.0)	
Moderately anxious/depressed	9 (60.0)	6 (40.0)	15 (100.0)	
Severely anxious/depressed	2 (50.0)	2 (50.0)	4 (100.0)	
Extremely anxious/depressed	0 (0.0)	1 (100.0)	1 (100.0)	
**Total**				
**Count**	43	27	70	
**% within Gender**	100.0	100.0	100.0	

Supplementary Table 4. *Cross-tabulation of EQ-5D dimensions (Mobility, Self-care, Usual Activities, Pain/Discomfort, Anxiety/Depression) according to % Total Body Surface Area (%TBSA). The table presents participants' distribution (count and percentage) with varying levels of TBSA (1-10%, 11-20%, 21-30%, 31-40%, 41-50%, 51-60%) reporting levels 1 to 5 for each dimension. Percentages are calculated within each %TBSA category.*

**EQ-5D**	**%TBSA**							
**Dimension**	**1-10**	**11-20**	**21-30**	**31-40**	**41-50**	**51-60**	**Total**	**p-value**
**Mobility N/(%)**								
No problems	39 (61.9)	15 (23.8)	4 (6.3)	2 (3.2)	2 (3.2.0)	1 (1.6)	63 (100.0)	0.141
Slight problems	2 (50.0)	1 (25.0)	1 (25.0)	0 (0.0)	0 (0.0)	0 (0.0)	4 (100.0)	
Moderate problems	0 (0.0)	0 (0.0)	2 (100.0)	0 (0.0)	0 (0.0)	0 (0.0)	2 (100.0)	
Severe problems	1 (100.0)	0 (0.0)	0 (0.0)	0 (0.0)	0 (0.0)	0 (0.0)	1 (100.0)	
Unable to	0 (0.0)	0 (0.0)	0 (0.0)	0 (0.0)	0 (0.0)	0 (0.0)	0 (0.0)	
**Self-care N/(%)**								
No problems	37 (64.9)	13 (22.8)	4 (7.4)	1 (1.8)	1 (1.8)	1 (1.8)	57 (100.0)	0.134
Slight problems	1 (14.3)	3 (42.9)	2 (28.6)	0 (0.0)	1 (14.3)	0 (0.0)	7 (100.0)	
Moderate problems	2 (50.0)	0 (0.0)	1 (25.0)	1 (25.0)	0 (0.0)	0 (0.0)	4 (100.0)	
Severe problems	2 (100.0)	0 (0.0)	0 (0.0)	0 (0.0)	0 (0.0)	0 (0.0)	2 (100.0)	
Unable to	0 (0.0)	0 (0.0)	0 (0.0)	0 (0.0)	0 (0.0)	0 (0.0)	0 (0.0)	
**Usual activities N/(%)**								
No problems	34 (65.4)	12 (23.1)	3 (5.8)	1 (1.9)	1 (1.9)	1 (1.9)	52 (100.0)	0.280
Slight problems	3 (37.5)	3 (37.5)	2 (25.0)	0 (0.0)	0 (0.0)	0 (0.0)	8 (100.0)	
Moderate problems	2 (28.6)	1 (14.3)	2 (28.6)	1 (14.3)	1 (14.3)	0 (0.0)	7 (100.0)	
Severe problems	3 (100.0)	0 (0.0)	0 (0.0)	0 (0.0)	0 (0.0)	0 (0.0)	3 (100.0)	
Unable to	0 (0.0)	0 (0.0)	0 (0.0)	0 (0.0)	0 (0.0)	0 (0.0)	0 (100.0)	
**Pain/ Discomfort N/(%)**								
No pain	25 (65.8)	8 (21.1)	4 (10.5)	0 (0.0)	1 (2.6)	0 (0.0)	38 (100.0)	0.475
Slight pain	12 (60.0)	6 (30.0)	1 (5.0)	1 (5.0)	0 (0.0)	0 (0.0)	20 (100.0)	
Moderate pain	2 (22.8)	2 (22.2)	2 (22.2)	1 (11.1)	1 (11.1)	1 (11.1)	9 (100.0)	
Severe pain	2 (100.0)	0 (0.0)	0 (0.0)	0 (0.0)	0 (0.0)	0 (0.0)	2 (100.0)	
Extremely pain	1 (100.0)	0 (0.0)	0 (0.0)	0 (0.0)	0 (0.0)	0 (0.0)	1 (100.0)	
**Anxiety/Depression N/(%)**								
Non anxious/depressed	19 (55.9)	9 (26.5)	4 (11.8)	0 (0.0)	1 (2.9)	1 (2.9)	34 (100.0)	0.247
Slight anxious/depressed	10 (62.5)	5 (31.3)	1 (6.3)	0 (0.0)	0 (0.0)	0 (0.0)	16 (100.0)	
Moderately anxious/depressed	10 (66.7)	2 (13.3)	1 (6.7)	1 (6.7)	1 (6.7)	0 (0.00)	15 (100.0)	
Severely anxious/depressed	3 (75.0)	0 (0.0)	0 (0.0)	1 (25.0)	0 (0.0)	0 (0.0)	4 (100.0)	
Extremely anxious/depressed	0 (0.0)	0 (0.0)	1 (100.0)	0 (0.0)	0 (0.0)	0 (0.0)	1 (100.0)	
**Total**								
**Count**	42	16	7	2	2	1	70	
**% within %TBSA**	100.0	100.0	100.0	100.0	100.0	100.0	100.0	

Supplementary Table 5. *Cross-tabulation of EQ-5D dimensions (Mobility, Self-care, Usual Activities, Pain/Discomfort, Anxiety/Depression) according to Degree of Burn. The table presents participants' distribution (number and percentage) with superficial, partial, full, superficial & partial, and partial & full-thickness burns, reporting levels 1 to 5 for each dimension. The distribution of responses is shown across each burn severity category, with p-values highlighting statistically significant differences in Mobility (p = 0.046) and Usual Activities (p = 0.037) between burn groups.*

**EQ-5D**	**Degree of burn**				
**Dimension**	**Superficial**	**Partial**	**Full**	**Total**	**p-value**
**Mobility N/(%)**					
No problems	1 (1.6)	39 (61.9)	23 (36.5)	63 (100.0)	0.046*
Slight problems	1 (25.0)	0 (0.0)	3 (75.0)	4 (100.0)	
Moderate problems	0 (0.0)	1 (50.0)	1 (50.0)	2 (100.0)	
Severe problems	0 (0.0)	0 (0.0)	1 (100.0)	1 (100.0)	
Unable to	0 (0.0)	0 (0.0)	0 (0.0)	0 (0.0)	
**Self-care N/(%)**					
No problems	1 (1.8)	35 (61.4)	21 (36.8)	57 (100.0)	0.183
Slight problems	1 (14.3)	2 (28.6)	4 (57.1)	7 (100.0)	
Moderate problems	0 (0.0)	3 (75.0)	1 (25.0)	4 (100.0)	
Severe problems	0 (0.0)	0 (0.0)	2 (100.0)	2 (100.0)	
Unable to	0 (0.0)	0 (0.0)	0 (0.0)	0 (0.0)	
**Usual activities N/(%)**					
No problems	1 (1.9)	32 (65.4)	17 (32.7)	52 (100.0)	0.037*
Slight problems	0 (0.0)	5 (62.5)	3 (37.5)	8 (100.0)	
Moderate problems	1 (14.3)	1 (14.3)	5 (71.4)	7 (100.0)	
Severe problems	0 (0.0)	0 (0.0)	3 (100.0)	3 (100.0)	
Unable to	0 (0.0)	0 (0.0)	0 (0.0)	0 (0.0)	
**Pain/Discomfort N/(%)**					
No pain	1 (2.6)	27 (71.1)	10 (26.3)	38 (100.0)	0.219
Slight pain	1 (5.0)	10 (50.0)	9 (45.0)	20 (100.0)	
Moderate pain	0 (0.0)	2 (22.2)	7 (77.8)	9 (100.0)	
Severe pain	0 (0.0)	1 (50.0)	1 (50.0)	2 (100.0)	
Extremely pain	0 (0.0)	0 (0.0)	1 (100.0)	1 (100.0)	
**Depression/Anxiety N/(%)**					
Non anxious/depressed	1 (2.9)	22 (64.7)	11 (32.4)	34 (100.0)	0.168
Slight anxious/depressed	1 (6.3)	11 (68.8)	4 (25.0)	16 (100.0)	
Moderately anxious/depressed	0 (0.0)	7 (46.7)	8 (53.3)	15 (100.0)	
Severely anxious/depressed	0 (0.0)	0 (0.0)	4 (100.0)	4 (100.0)	
Extremely anxious/depressed	0 (0.0)	0 (0.0)	1 (100.0)	1 (100.0)	
**Total**					
**Count**	2	40	28	70	
**% within Degree of Burn**	100.0	100.0	100.0	100.0	

*A p-value <0.05 was considered as significant.
